# Checklist of the smaller families of Opomyzoidea, Anthomyzidae, Asteiidae, Aulacigastridae, Clusiidae, Odiniidae, Opomyzidae and Periscelididae (Diptera) of Finland

**DOI:** 10.3897/zookeys.441.7141

**Published:** 2014-09-19

**Authors:** Jere Kahanpää

**Affiliations:** 1 Finnish Museum of Natural History, Zoology Unit, P.O. Box 17, FI–00014 University of Helsinki, Finland

**Keywords:** Checklist, Finland, Diptera, biodiversity, faunistics

## Abstract

A species checklist is presented for Finland covering seven smaller families of Opomyzoidea: Anthomyzidae, Asteiidae, Aulacigastridae, Clusiidae, Odiniidae, Opomyzidae and Periscelididae (Diptera).

## Introduction

The superfamily Opomyzoidea is likely to be para- or polyphyletic (see Wiegmann et al. 2011). It has long been used as a dumping grounds for acalyptrate taxa that do not fit well elsewhere. As such, the superfamily is poorly defined and hardly any two authors agree on its definition. Opomyzoidea includes one large family (Agromyzidae, the leaf-mining flies, with over 3000 described species) and more than a dozen small families. The exact number of recognized families varies. The Agromyzidae are treated elsewhere in this volume of ZooKeys. The remaining opomyzoid families are presented here in alphabetical order.

Hackman (1980) last listed the Finnish species of these families. World catalogues have recently been published for Periscelididae (Mathis and Rung 2011) and Odiniidae (Gaimari and Mathis 2011). For Anthomyzidae the Revision of West Palearctic Anthomyzidae by Roháček (2006, 2009) is followed. Three of the four Palearctic *Aulacigaster* species have been found in Europe (Kassebeer 2001, Rung and Mathis 2011). Papp and Withers (2011) reviewed the Palearctic Periscelidinae and proposed some nomenclatorial changes. Hackman’s (1959) review of the Finnish opomyzid fauna is still valuable but several additional species have since been recognized.

**Table 1.** Number of species by family.

Family	Number of species in			Level of knowledge
	World (Pape et al. 2011)	Europe	Finland	
Anthomyzidae	93	28	15	average
Asteiidae	136	18	6	average
Aulacigastridae	18	4	2	good
Clusiidae	360	14	12	good
Odiniidae	64	14	5	average
Opomyzidae	59	33	16	average
Periscelididae	83	6	4	average

## Checklist

suborder Brachycera Macquart, 1834

clade Eremoneura Lameere, 1906

clade Cyclorrhapha Brauer, 1863

infraorder Schizophora Becher, 1882

clade Muscaria Enderlein, 1936

parvorder Acalyptratae Macquart, 1835

superfamily Opomyzoidea Fallén, 1820

**ANTHOMYZIDAE** Czerny, 1903

***ANTHOMYZA*** Fallén, 1810

*Anthomyza collini* Andersson, 1976

*Anthomyza dissors* Collin, 1944

*Anthomyza elbergi* Andersson, 1976

= *sordidella* auct. nec (Zetterstedt, 1848)

*Anthomyza gracilis* Fallén, 1823

*Anthomyza macra* Czerny, 1928

*Anthomyza pallida* (Zetterstedt, 1838)

= *unguicella* (Zetterstedt, 1838)

*Anthomyza paraneglecta* Elberg, 1968

*Anthomyza pleuralis* Czerny, 1928

***ARGANTHOMYZA*** Roháček, 2009

*Arganthomyza socculata* (Zetterstedt, 1847)

= *ungulata* (Loew, 1873)

***CAREXOMYZA*** Roháček, 2009

*Carexomyza caricis* (Roháček, 1999)

***FUNGOMYZA*** Roháček, 1999

*Fungomyza albimana* (Meigen, 1830)

***PARANTHOMYZA*** Czerny, 1902

*Paranthomyza nitida* (Meigen, 1838)

***STIPHROSOMA*** Czerny, 1928

= ***Ptenotaenia*** Enderlein, 1936

*Stiphrosoma laetum* (Meigen, 1830)

= *cingulatum* misid.

*Stiphrosoma sabulosum* (Haliday, 1837)

***TYPHAMYZA*** Roháček, 1992

*Typhamyza bifasciata* (Wood, 1911)

**ASTEIIDAE** Rondani, 1856

ASTEIINAE Rondani, 1856

***ASTEIA*** Meigen, 1830

**sg. *Asteia*** Meigen, 1830

*Asteia amoena* Meigen, 1830

*Asteia concinna* Meigen, 1830

*Asteia elegantula* Zetterstedt, 1847

SIGALOESSINAE Sabrosky, 1956

***LEIOMYZA*** Macquart, 1835

*Leiomyza dudai* Sabrosky, 1956

*Leiomyza laevigata* (Meigen, 1830)

*Leiomyza scatophagina* (Fallén, 1823)

**AULACIGASTRIDAE** Duda, 1924

***AULACIGASTER*** Macquart, 1835

= ***Aulacogaster*** emend.

*Aulacigaster leucopeza* (Meigen, 1830)

*Aulacigaster pappi* Kassebeer, 2001

**CLUSIIDAE** Handlirsch, 1884

CLUSIINAE Handlirsch, 1884

***CLUSIA*** Haliday, 1838

= ***Paraclusia*** Czerny, 1903

*Clusia flava* (Meigen, 1830)

*Clusia tigrina* (Fallén, 1820)

CLUSIODINAE

***CLUSIODES*** Coquillett, 1904

= ***Heteroneura*** Fallén, 1823 preocc.

*Clusiodes albimanus* (Meigen, 1830)

*Clusiodes apicalis* (Zetterstedt, 1848)

*Clusiodes caledonicus* (Collin, 1912)

= *gentilis* misid.

*Clusiodes freyi* Tuomikoski, 1933

*Clusiodes geomyzinus* (Fallén, 1823)

*Clusiodes microcercus* Stackelberg, 1955

*Clusiodes pictipes* (Zetterstedt, 1855)

*Clusiodes ruficollis* (Meigen, 1830)

*Clusiodes verticalis* (Collin, 1912)

***HENDELIA*** Czerny, 1903

*Hendelia beckeri* Czerny, 1903

**ODINIIDAE** Hendel, 1920

ODINIINAE Hendel, 1920

***NEOALTICOMERUS*** Hendel, 1903

*Neoalticomerus formosus* (Loew, 1844)

***ODINIA*** Robineau-Desvoidy, 1830

*Odinia boletina* (Zetterstedt, 1848)

*Odinia czernyi* Collin, 1952

= *meijerei* misid.

*Odinia ornata* (Zetterstedt, 1838)

*Odinia xanthocera* Collin, 1952

**OPOMYZIDAE** Fallén, 1820

***ANOMALOCHAETA*** Frey, 1921

*Anomalochaeta guttipennis* (Zetterstedt, 1838)

***GEOMYZA*** Fallén, 1810

*Geomyza angustipennis* Zetterstedt, 1847

*Geomyza apicalis* (Meigen, 1830)

*Geomyza consobrina* Zetterstedt, 1847

*Geomyza hackmani* Nartshuk, 1984

= *combinata* auct. nec (Linnaeus, 1767)

= *balachowskyi* misid.

*Geomyza majuscula* (Loew, 1864)

= *breviforceps* Hackman, 1959

*Geomyza martineki* Drake, 1992

*Geomyza paganettii* (Strobl, 1909)

*Geomyza pilosula* Czerny, 1928

*Geomyza tripunctata* Fallén, 1823

***OPOMYZA*** Fallén, 1820

*Opomyza florum* (Fabricius, 1794)

*Opomyza germinationis* (Linnaeus, 1758)

*Opomyza lineatopunctata* von Roser, 1840

*Opomyza nigriventris* Loew, 1865

*Opomyza punctata* Haliday, 1833

*Opomyza punctella* Fallén, 1820

**PERISCELIDIDAE** Oldenberg, 1914

PERISCELIDINAE Oldenberg, 1914

***MYODRIS*** Lioy, 1864

= ***Microperiscelis*** Oldenberg, 1914

*Myodris annulata* (Fallén, 1813)

***PERISCELIS*** Loew, 1858

*Periscelis annulipes* Loew, 1858

*Periscelis nigra* (Zetterstedt, 1860)

*Periscelis winnertzii* Egger, 1862

## Excluded species

*Clusiodes gentilis* (Collin, 1912) misidentified

*Geomyza balachowskyi* Mesnil, 1934 misidentified

*Odinia meijerei* Collin, 1952 misidentified

## Notes

***Aulacigaster pappi* Kassebeer, 2001**. Most of the Finnish *Aulacigaster* material belong to this species, including all specimens currently stored in the Finnish Museum on Natural History (MZH). Storå (1956, 1957) and Hackman (1980) misidentified *Aulacigaster pappi* as *Aulacigaster leucopeza*. A few Finnish specimens of the real *Aulacigaster leucopeza* were found in private collections.

***Clusiodes gentilis* (Collin, 1912)**. Lonsdale and Marshall (2007) record this species from Finland on a map. The data is apparently taken from Frey (1928). As Tuomikoski (1936) has pointed out, Frey misidentified *Clusiodes caledonicus* as *Clusiodes gentilis*.
